# Discovery of novel fragments inhibiting O-acetylserine sulphhydrylase by combining scaffold hopping and ligand–based drug design

**DOI:** 10.1080/14756366.2018.1512596

**Published:** 2018-09-17

**Authors:** Joana Magalhães, Nina Franko, Giannamaria Annunziato, Martin Welch, Stephen K. Dolan, Agostino Bruno, Andrea Mozzarelli, Stefano Armao, Aigars Jirgensons, Marco Pieroni, Gabriele Costantino, Barbara Campanini

**Affiliations:** a P4T group, Department of Food and Drug, University of Parma, Parma, Italy;; b Laboratory of Biochemistry and Molecular Biology, Department of Food and Drug, University of Parma, Parma, Italy;; c Department of Biochemistry, Cambridge University, Cambridge, United Kingdom;; d Experimental Therapeutics Program, IFOM – The FIRC Institute for Molecular Oncology Foundation, Milano, Italy;; e National Institute of Biostructures and Biosystems, Rome, Italy;; f Institute of Biophysics, CNR, Pisa, Italy;; g Centro Interdipartimentale “Biopharmanet-tec”, Università degli Studi di Parma, Parma, Italy;; h Latvian Institute of Organic Synthesis, Riga, Latvia;; i Centro Interdipartimentale Misure (CIM)’G. Casnati’, University of Parma, Parma, Italy

**Keywords:** Scaffold hopping, fragments, O-acetylserine sulphhydrylase, medicinal chemistry, pyrazoles, ligand-based drug design

## Abstract

Several bacteria rely on the reductive sulphur assimilation pathway, absent in mammals, to synthesise cysteine. Reduction of virulence and decrease in antibiotic resistance have already been associated with mutations on the genes that codify cysteine biosynthetic enzymes. Therefore, inhibition of cysteine biosynthesis has emerged as a promising strategy to find new potential agents for the treatment of bacterial infection. Following our previous efforts to explore OASS inhibition and to expand and diversify our library, a scaffold hopping approach was carried out, with the aim of identifying a novel fragment for further development. This novel chemical tool, endowed with favourable pharmacological characteristics, was successfully developed, and a preliminary Structure–Activity Relationship investigation was carried out.

## Introduction

Since the 1940s, antibacterial agents have greatly reduced illness and death from infectious diseases. However, the misuse of these drugs resulted in adaptation of the bacteria to their killing action, and nowadays antibiotics are less effective with a few of them being useless (“About Antimicrobial Resistance | Antibiotic/Antimicrobial Resistance | CDC,” 2018; “WHO | Antimicrobial resistance,” 2018). Therefore, it is necessary to adopt novel strategies to fight antibacterial resistance. Cysteine *de novo* biosynthesis, present only in bacteria and plants, converts inorganic sulphur into cysteine, a precursor of several important biomolecules such as methionine, Fe-S clusters, thiamin, glutathione, and biotin^1^^,^^2^.

The reductive sulphur assimilation pathway involves five enzymes that reduce sulphate to bisulfide and is connected with the serine activation reaction, where L-serine is converted to *O*-acetylserine (OAS) by serine acetyltransferase (SAT). *O*-acetylserine sulfhydrylase (OASS) catalyzes the β-replacement of the acetoxy group of OAS by bisulphide, generating cysteine^3^^,^^4^. OASS is a member of the cysteine synthase superfamily, and it is a pyridoxal 5’-phosphate (PLP)-dependent enzyme that exists in two isoforms: OASS-A and OASS-B. Both isozymes were firstly identified and characterised in *Salmonella enterica* serovar Typhimurium (*Salmonella*), although the specific role of OASS-B is still debated^5^. OAS is the preferred substrate for both isoforms but while bisulfide is the only sulfur source used by OASS-A, OASS-B can use both bisulphide and thiosulphate^6^^,^^7^. The three-dimensional structures^8–21^ of OASS-A and OASS-B isoforms has shown that the active site is in a cleft between the domains, where a PLP molecule is covalently bound to a conserved lysine residue. SAT and OASS-A combine in a tight molecular complex that is called the cysteine synthase complex, and the SAT C-terminal oligopeptide shows OASS competitive inhibitory properties. On the other hand, OASS-B does not interact with SAT, which suggests a different regulatory role in the reductive sulphur assimilation pathway^3^^,^^4^^,^^22^.

**Figure 1.**
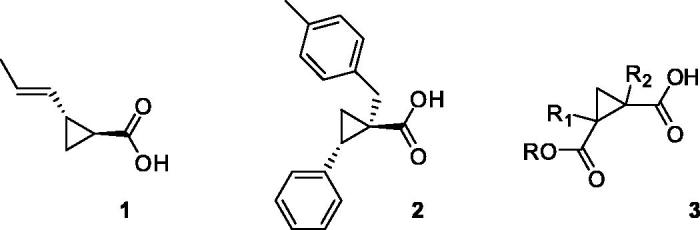
Chemical structure of the previously identified OASS inhibitors.

Over the years, inhibitors of both OASS-A and B isoforms have been developed. Our group was particularly successful in preparing peptidic^23^^,^^24^ and non-peptidic inhibitors^22^^,^^25–28^ by rational design inspired by the structure of SAT C-terminal sequence. Among the many structure–activity relationship (SAR) hints considered, the carboxylic moiety of Ile267 of SAT was found out to be essential for the interaction with the target enzyme, and the inhibitors prepared could not be devoid of this moiety^23^. Within the set of synthetic inhibitors with potencies ranging from low millimolar to low micromolar, (±)-trans-2-[(1E)-prop-1-en-1-yl]cyclopropanecarboxylic acid (**1**, Figure 1), with a K_d_ of 1.46 μM^25^, was selected for further optimization as it suffered from several weaknesses, such as poor chemical feasibility and stability.

Therefore, combining a structure-based and ligand-based drug design approach, and with the help of spectroscopic methods such as STD-NMR, we planned and synthesised a series of cyclopropanecarboxylic acid derivatives that bind to both OASS isoforms at nanomolar concentrations, as in the case of (1*S*,2*S*)-1–(4-methylbenzyl)-2-phenylcyclopropanecarboxylic acid (**2**, Figure 1)^28^. Despite the remarkably high affinity (K_d_*_-OASS_* = 0.028 μM), this compound did not show any activity when tested against *Salmonella in vitro*. We were able to demonstrate (unpublished data) that the lack of efficacy was due to the poor permeability of this derivative through the cell envelope, that in the case of Gram-negative bacteria poses particular challenges^29^. Although there are no simple rules for predicting Gram-negative bacterial entry, it is known that molecules such as quinolones, that have negative ClogP and polar character, are able to accumulate in Gram-negative bacteria, whereas high-size molecules such as macrolides are not active towards Gram-negative because of the bigger size^30–32^. Keeping in mind these considerations, we have tried to modify the structure of compound **2**, that has high potency but unfavourable physicochemical characteristics (ClogP = 3.60), with a focused medicinal chemistry campaign. In a first attempt^26^ we have reported the design and synthesis of a series of cyclopropane-1,2-dicarboxylic acids variously functionalized (**3**, Figure 1), with the aim of enhancing the polar character of the molecules; the carboxylic moiety was maintained, as well as the cyclopropane scaffold. Unfortunately, the binding properties of these novel analogues could not be improved, and also in this case, the most active derivative failed to show activity in bacterial cells (data not shown). Therefore, we focused our attention toward the design and synthesis of molecules of smaller size and lower ClogP. We deemed essential to maintain the carboxylic moiety, as its interactions with the OASS active site have shown to be pivotal for molecular binding. At the same time, we wanted to bypass the cyclopropane core, which poses synthetic challenges and restrictions in particular with regard to the stereochemistry of the derivatives. To this end, we carried out a scaffold hopping approach leading to the discovery of a novel fragment, which was further investigated to optimize its affinity toward the target enzyme. The computational approach leading to this fragment, and the preliminary structure-activity relationships study around it are herein reported.

## Methods

### Scaffold hopping

Compound **2** (Figure 1) was docked on the chain A of *Salmonella* crystal structure (pdb code 1OAS) using the LeadIt software. Protein preparation was performed using Yazara and LeadIT tools and the amino acid residues Asn70 and Gln143 were flipped while Thr73 was rotated through a 90° angle. In order to preserve the interactions of the carboxylic acid group, the pharmacophore Gln143 and Thr73 was applied. Ligand preparation was performed on Chemaxon software, Marvin. Afterward, using the ReCore tool of LeadIt software and keeping the tolyl and the carboxylic moieties of the original hit, an alternative scaffold to the cyclopropane of compound **2** was searched in Zinc database.

### Synthetic chemistry

**Scheme 1.**
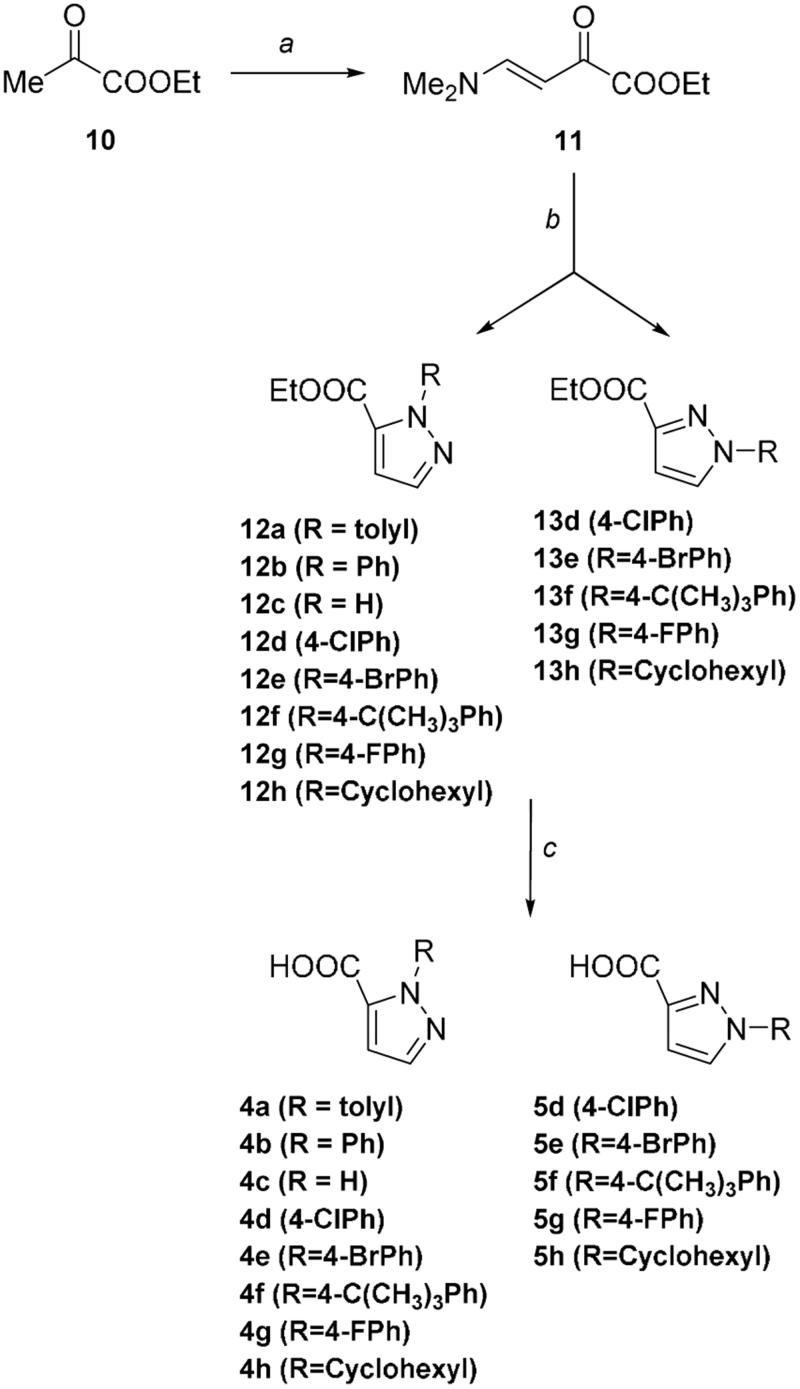
Synthetic scheme to obtain the title compounds **4a**–**h** and **5d**–**h**. Reagents and Conditions: (a) Me_2_NCH(OEt)_2_, CH_2_Cl_2_, rt; (b) R-NH_2_NH_2_, MeOH, 20–60 °C; (c) NaOH, EtOH, 80 °C.

With the exception of compounds **14**–**16**, commercially available, pyrazole derivatives were synthesized according to a protocol already reported for the preparation of heterocyclic compounds^33^. This procedure was deemed of interest because of its lack of regiospecificity, that allowed us to obtain simultaneously both the regioisomers, which could be easily isolated with flash chromatography. Ethyl (*E*)-4-(dimethylamino)-2-oxobut-3-enoate **11** was prepared from commercially available ethyl pyruvate **10**, that reacted with dimethylformamide diethyl acetal in dichloromethane at room temperature (Scheme 1). Reacting **11** with the properly substituted hydrazine hydrochloride in methanol afforded both the 1-arylpyrazole-3-carboxylic acid ethyl esters **12a**–**h** and the 1-arylpyrazole-5-carboxylic acid ethyl ester isomers **13d**–**h**, which were therefore hydrolyzed to obtain the desired title compounds **4a**–**h** and **5d**–**h.**

Enaminone **11** exists in solution in CDCl_3_ as a single isomer, the *trans*-orientated nuclei since the magnitude of the coupling constant (*J* = 12.5 Hz). As anticipated, cyclocondensation of compound **11** with the properly substituted hydrazine hydrochloride afforded both the pyrazole carboxylates **12** and the regioisomers **13**.

**Figure 2.**
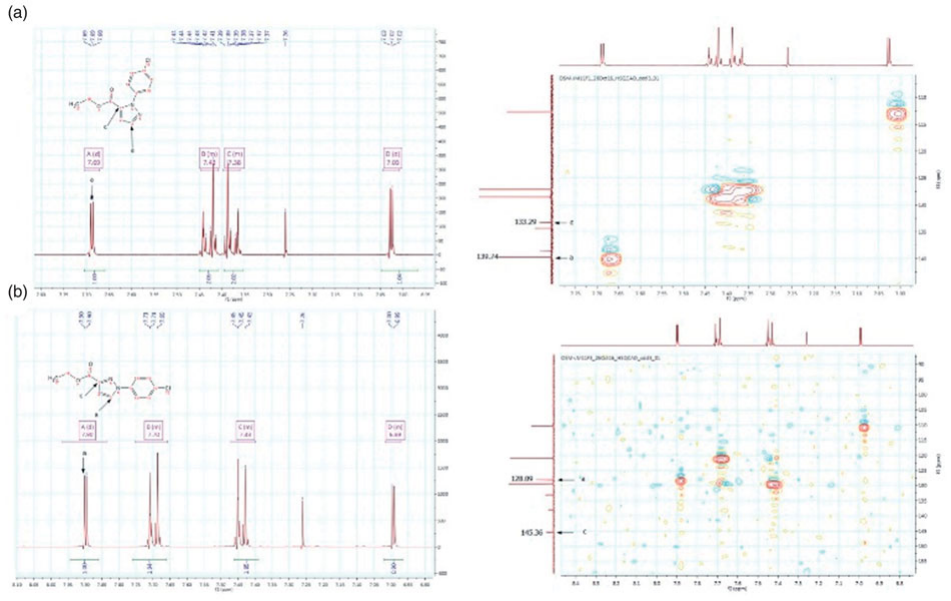
Enlargement of the ^1^H NMR and HMQC NMR of compounds 12d (a) and **13d** (b).

Pyrazole carboxylate **12**, which corresponds to the 1,5-isomer, is the product obtained in higher yield. The reason of this regioselectivity might be due to the fact that the substituted hydrazine attacks the enaminone preferentially through its primary amino group instead of the secondary one, as the latter is more hindered and, in general, less nucleophilic. Curiously, in only one case (**12a**), the cyclocondensation reaction afforded the single 1-arylpyrazole-5-carboxylate isomer. Although in agreement with the hypothesis above reported, stereoelectronic reasons might not be sufficient to explain this result, since the 4-*t*-butyl phenylhydrazine allows to obtain both the regioisomers. Therefore, to better explain the selectivity of the reaction, more information will be furnished by the expansion of the series with a higher number of substituted hydrazines. The presence and the identification of the two regioisomers (Figure 2) was investigated by NMR analysis since the proton at C_a_ in compound **13** is de-shielded compared with that in compound **12**. Moreover, the 1,3 and 1,5 isomers also present characteristic^13^ C spectra where the C_a_ and C_c_ present different chemical shields, being *C*_a_ more shielded and *C*_c_ located upfield in the case of regioisomer **13** versus regioisomer **12** (Figure 2). In order to refine this conformational analysis, we also carried out HMQC-NMR experiments (Figure 2).

All of the compounds prepared were then hydrolysed by means of aqueous sodium hydroxide to afford the carboxylic group in good yields.

### Protein preparation

The genes *cysK* and *cysM* encoding STOASS-A and STOASS-B, respectively, were subcloned from pET16b^34^ to pET19m in order to replace the restriction site for Factor Xa with the one for TEV protease. pET19m-*STcysK* and pET19m-*STcysM* were transformed in E. coli Rosetta^TM^ (DE3) competent cells for protein expression. Protein expression and purification was performed following published protocols^34^ with some modifications. Briefly, protein expression was induced on bacterial growth in exponential phase by IPTG for 4 and 5 h for OASS-B and OASS-A, respectively. Cells were lysed by pulsed sonication in 50 mM NaP, 300 mM NaCl, pH 7, with the addition of 1 mg/mL lysozyme. Purification was performed by immobilized metal affinity column (IMAC) on Co^2+^ ions (Talon™, Clontech Laboratories, Inc., Mountain View, CA). Proteins were eluted with 250 mM (His_6_-OASS-A) or 600 mM (His_6_-OASS-B) imidazol. His-tag was removed from His_6_-OASS-A by treatment with TEV protease during O/N dialysis in 10 mM HEPES pH 8. His-tag was removed from His_6_-OASS-B by treatment with TEV protease in 20 mM HEPES, 100 mM NaCl, 1 mM TCEP pH 8 for 24 h at 5 °C. Tag and undigested proteins were removed with batch IMAC, yielding more than 95% pure protein based on SDS-PAGE analysis. OASS-A was stored in 10 mM HEPES pH 8 and OASS-B in 20 mM HEPES, 100 mM NaCl, 1 mM TCEP pH 8.

### Activity and binding assay

The potency of the compounds was screened at two fixed concentrations (1 μM and 1 mM) by a 96-well plate-adapted activity assay based on the reaction of cysteine with ninhydrin under acidic conditions^28^^,^^35^. The concentration of bisulfide was saturating (600 μM), whereas the concentration of OAS was set at Km to increase the sensitivity. The dissociation constant of selected compounds for StOASS-A and OASS-B was measured by a fluorimetric method published elsewhere^23^^,^^24^. Briefly, a solution containing 1 µM StOASS-A in 100 mM Hepes pH 7 was titrated with increasing concentrations of compound at 20 °C. The fluorescence emission intensity of the PLP cofactor at 505 nm upon excitation at 412 nm was collected after each addition, subtracted by the blank and normalized by the protein dilution. The dependence of the emission intensity at 505 nm on the concentration of the compound was fitted to a binding isotherm^23^ to calculate the dissociation constant of the protein-ligand complex. As demonstrated elsewhere^25^^,^^28^, the fluorimetric method allows the calculation of the intrinsic dissociation constant of a competitive inhibitor for the enzyme and the calculated *K*_d_ is in very good agreement with the IC_50_ measured by activity assays.

## Results and discussion

**Figure 3.**
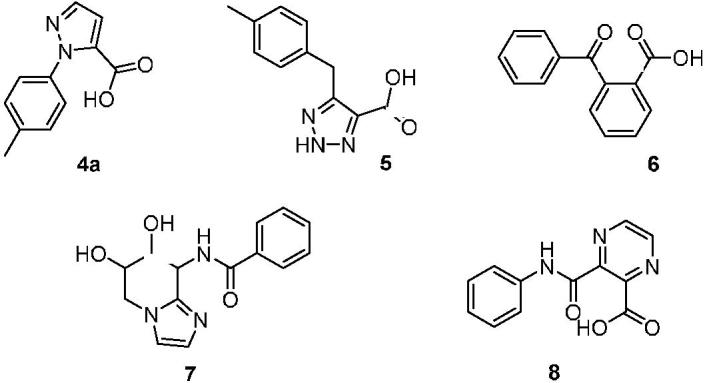
Putative hits obtained after scaffold hopping of the previously identified compound **2**.

A scaffold hopping using a hit compound previously identified by us was performed in order to identify a novel inhibitor of OASS to be further investigated. Our group has already made use of computational tools in order to broaden the body of SAR of these derivatives^36^. Among the several scaffolds suggested by the program (Figure 3), we deemed of particular interest to further investigate hit compound **4a**, which was characterised by an *N*-substituted pyrazole central spacer.

**Figure 4.**
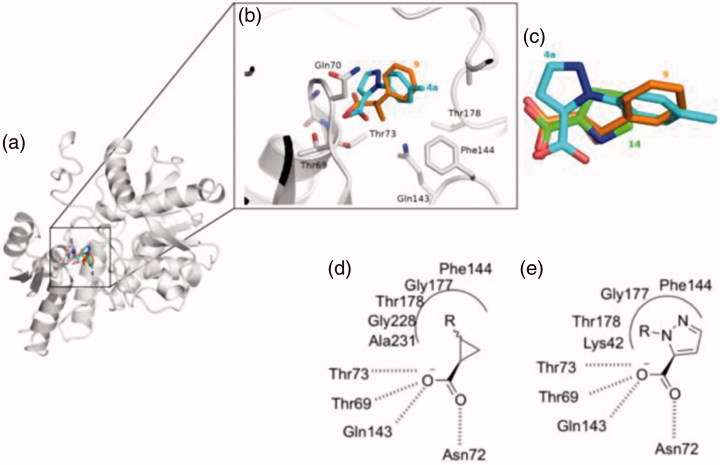
Docking results of compound **4a** into StOASS-A binding site. (a) StOASS-A (white cartoon and sticks) in complex with compound **9** (orange sticks) and **4a** (cyan sticks); (b) StOASS-A active site (white cartoon and sticks) in complex with compound **9** (orange sticks) and **4a** (cyan sticks); (c) alignment of the binding mode of **9**, **4a** and **14** highlighting how the compounds accommodate the pharmacophoric features in similar directions; (d) bi-dimensional representation of interactions of StOASS-A with the cyclopropane scaffold (e) and with compound **4a** (c).

This is due to several considerations. First of all, the analysis of the key interactions established by compound **4a** with OASS backbone (Figure 4) anticipates a similar binding pattern as for our previously reported hits^25^^,^^27^^,^^36^.

**Figure 5.**
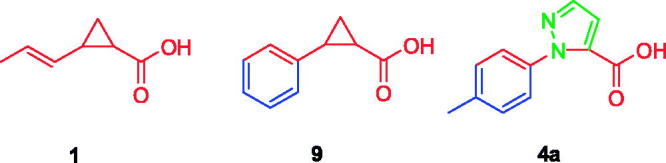
Chemical structure of the previously reported OASS inhibitors **1**, **9** and compound **4a**.

After visual inspection, it was possible to envisage also a structural similarity of **4a** when superimposed with some of our previously reported derivatives bearing a cyclopropane scaffold^28^, such as, for instance, compound **9** (Figure 5).

Moreover, compared to compound **9** and to the most active derivative compound **2**, molecule **4a** has a higher total polar surface area (TPSA) and comparable ClogP, anticipating improved drug-like properties. Finally, we considered the synthetic feasibility as another advantage, with the possibility to add substituents at the pyrazole core, or to further manipulate the structure with other heterocycles. This compound was thus synthesized and evaluated for its capability to bind and inhibit the enzymatic activity for the two OASS isoforms at two concentrations. At 1 mM compound **4a** showed a 50% inhibition of OASS-A activity and a similar value was found also for OASS-B (Table 1). Although not remarkable, this result was considered worth of further investigation, and prompted us to prepare a number of derivatives. As a preliminary analysis, we wanted to check which was the most favourable attachment point for the phenyl ring (N-1 or N-2), whether the aromatic ring could be adorned with small electron-withdrawing groups (EWG) or electron-donating groups (EDG), whether the aromatic ring could be substituted with a cycloaliphatic structure and, finally, whether the small fragment-like pyrazolecarboxylic acid alone was active. First, we investigated the 1-arylpyrazole-3-carboxylic acid and the 1-arylpyrazole-5-carboxylic acid regioisomers, with the phenyl ring either unsubstituted or adorned with small functional groups. We took advantage from a straight synthetic protocol based on a non-regiospecific reaction, which allowed the isolation of the two isomers starting from common starting materials (see Materials and Methods section). We then checked the effect of a substituent at the phenyl ring, which was arbitrarily introduced at the *para* position in this preliminary round of modifications. In the cases of small EWGs attached at the phenyl ring, not any difference in the potency could be noticed for compounds **4d** vs **5d** and **4 g** vs **5 g** (Table 1). A bulkier substituent such as the bromine remarkably favours the 1,5-isomer compared to the 1,3-isomer (**4e** vs **5e**, Table 1), giving a > 80% inhibition on OASS-A at 1 mM. An opposite pattern is noticed when bulky EDGs such as the *tert*-Butyl are introduced at the phenyl ring, as in this case the 1,3-isomer resulted twofold more affine to the target enzyme than the corresponding 1,5-isomer (**4f** vs **5f**, Table 1). A smaller EDG such as the methyl does not affect the activity (**4f** vs **4a**, Table 1). Interestingly, substitution of the aromatic ring with a cyclohexane is still tolerated, with a percentage of inhibition similar to that of the derivatives bearing an aromatic substituent (see compounds **4 h** and **5h**).

**Table 1.** Inhibitory potency of pyrazoles on the activity of recombinant OASS from *Salmonella.*

Cpd^a^	% inhibition OASS-A^c^		% inhibition OASS-B^c^	
	1 µM	1 mM	1 µM	1 mM
**4a**	ns^b^	45 ± 3	ns	65 ± 2
**4b**	ns	66 ± 1	11 ± 3	65 ± 1
**4c**	9 ± 7	94 ± 1	ns	82 ± 1
**4d**	ns	60 ± 2	9 ± 6	56 ± 1
**4e**	ns	84 ± 1	ns	58 ± 2
**4f**	ns	47 ± 1	ns	52 ± 5
**4g**	8 ± 1	95 ± 1	14 ± 2	62 ± 1
**4h**	ns	57 ± 1	ns	30 ± 1
**5d**	ns	63 ± 1	15 ± 2	63 ± 1
**5e**	ns	62 ± 1	ns	35 ± 2
**5f**	ns	81 ± 1	11 ± 6	82 ± 1
**5g**	ns	73 ± 1	ns	36 ± 3
**5h**	ns	79 ± 1	ns	43 ± 3

a For structures see Scheme 1.

b Not Significant.

c All assays were performed in two replicates.

Despite the range of activity allowed only a rough SAR, these results somehow confirmed the presence of a lipophilic pocket in the proximity of the enzyme active site where the pyrazole substituent can locate, as previously noticed by us^27^^,^^36^^,^^37^. In addition, the activity shown by compounds **4e** and **5f** demonstrated that the size of the molecule may have some influence on the binding to the enzyme pocket. Since these considerations, we were very surprised that the 1H-pyrazole-5-carboxylic acid **(4c)** was found to be the most potent fragment-like hit of the series, with more than 90% inhibition at 1 mM and an experimental *K*_d_ of 122 μM to OASS-A and a *K*_d_ of 272 μM towards OASS-B (Table 2). It must be remembered that, as we have already demonstrated,^28^, in case of competitive inhibitors such as the compounds reported, *K*_d_ correspond to the *K*_i_. These findings lead to speculate that, although the presence of the lipophilic pocket has been predicted by computational studies and confirmed by the small set of compounds reported in this paper; however, its filling with lipophilic moieties such as aromatic rings seems to be detrimental for the binding. Further investigation is needed to better select the proper moieties that can interact more efficiently with this side-pocket.

**Table 2.** Overall properties of compounds 2 and 4a and dissociation constant plus ligand efficiency of compounds **13–15** determined for StOASS.

Cpd	Structure	MW (g/mol)	*K*_d_ OASS-A (µM)	*K*_d_ OASS-B (µM)	LE (OASS-A)^b^ kcal/mol/HA	LE (OASS-B)^b^ kcal/mol/HA	ClogP^c^	TPSA^c^
**2**		266.33	0.03^a^	0.049	0.52	0.50	3.60	37.30
**4c**		112.09	122 ± 5	272 ± 10	0.67	0.61	0.13	65.98
**14**		111.1	59 ± 11	465 ± 50	0.72	0.60	0.66	53.09
**15**		113.07	120 ± 12	548 ± 59	0.67	0.60	0.04	63.33
**16**		129.14	52 ± 6	338 ± 14	0.74	0.60	0.17	50.19

a When SD is not reported, it means that figures are meaningless due to rounding of the decimals.

b LE = (1.37/HA). pIC_50_ where HA refers to heavy atoms.

c ClogP and TPSA were calculated using molinspiration website (http://www.molinspiration.com/).

Along with the interesting binding properties, compound **4c** possessed chemical highly desirable characteristics known to be beneficial for Gram-negative penetration, such as the low ClogP, high TPSA and the reduced size^31^^,^^32^. Further analysis of the interactions established between this compound and OASS backbone encouraged us to explore other five-membered heterocycles functionalized with a carboxylic acid functional group, as it was considered crucial for the interaction at the enzyme active site (**14**–**16**, Table 2).

Surprisingly, all of the compounds tested were found to display interesting binding properties to StOASS-A, with a noticeable improvement in the affinity compared to the hit compound derived from scaffold hopping (**4a**), and also better than fragment **4c** (Table 2).

The activity shown by the 1H-pyrazole-5-carboxylic acid derivatives is considerably lower than that reported by the most active derivative **2** used as the reference compound. However, it must be considered that both the molecules have a similar value of ligand efficiency (LE). LE, calculated as described by Chen et al.^38^, is a parameter that serves as a valuable metric to analyse the binding of small molecules and fragment-like compounds and to assess the viability of a fragment as a starting point for optimisation. Therefore, together with other favourable drug-like characteristics^39–42^, the good LE of compounds **14** and **16** presents these fragment-like derivatives as suitable candidates to further explore in the search of novel OASS inhibitors to be used as antibacterial adjuvants.

## Experimental part

### General information

All the reagents and some final compounds (**14**–**16**) were purchased from Sigma-Aldrich, Alfa-Aesar, and TCI at reagent purity and, unless stated otherwise, were used without further purification. Reactions were monitored by thin-layer chromatography on silica gel-coated aluminium foils (silica gel on Al foils, SUPELCO Analytical, Sigma-Aldrich) at 254 and 365 nm. Where indicated, intermediates and final products were purified by silica gel flash chromatography (silica gel, 0.040−0.063 mm), using appropriate solvent mixtures. ^1^H NMR and^13^ C NMR spectra were recorded on a BRUKER AVANCE spectrometer at 300, 400 and 100 MHz, respectively, with TMS as internal standard. ^1^H NMR spectra are reported in this order: multiplicity and number of protons. Standard abbreviations indicating the multiplicity were used as follows: s = singlet, d = doublet, dd = doublet of doublets, t = triplet, q = quadruplet, m = multiplet, and br = broad signal. HPLC/MS experiments were performed with an Agilent 1100 series HPLC apparatus, equipped with a Waters Symmetry C18, 3.5 μm, 4.6 mm × 75 mm column and an MS: Applied Biosystem/MDS SCIEX instrument, with API 150EX ion source. All compounds were tested as 95% purity or higher (by HPLC/MS).

#### Ethyl (E)-4-(dimethylamino)-2-oxobut-3-enoate (11)

Ethyl pyruvate (17.22 mmol, 2g) was solubilised in dichloromethane (34 ml) and to the previous solution was added dropwise dimethylformamide dimethyl acetal (17.22 mmol, 2g). Reaction mixture was stirred at room temperature for 4 h and after the volatiles were evaporated under reduced pressure to obtain a dark brown oil that was purified by flash column chromatography using 1% MeOH in dichloromethane to obtain the product as a brown yellowish oil in 54% yield. ^1^H NMR (300 MHz, Chloroform-*d*) δ 7.83 (d, *J* = 12.5 Hz, 1H), 5.81 (d, *J* = 12.5 Hz, 1H), 4.30 (qd, *J* = 7.1, 0.9 Hz, 2H), 3.18 (s, 3H), 2.95 (s, 3H), 1.36 (td, *J* = 7.1, 0.9 Hz, 3H).

### General procedure for the synthesis of ethyl 1R-pyrazole-5-carboxylate

To a solution of ethyl (E)-4-(dimethylamino)-2-oxobut-3-enoate (130 mg, 0.76 mmol) in methanol (580 μl) was added the properly substituted hydrazine (0.76 mmol). Reaction mixture was stirred at 60 °C for 6 h. The volatiles were evaporated under reduced pressure and the residue was solubilized in chloroform, treated with a saturated solution of NaHCO_3_ and then extracted with chloroform. The combined organic layers were washed with brine and dried over anhydrous magnesium sulphate, filtrated and concentrated under reduced pressure.

#### Ethyl 1-(p-tolyl)-1H-pyrazole-5-carboxylate (Compound 12a)

The product was purified by flash chromatography on silica gel using 3% ethyl acetate in petroleum ether. The product was obtained as a light brown powder in 48% yield. ^1^H NMR (300 MHz, Chloroform-*d*) δ 7.67 (d, *J* = 1.9 Hz, 1H), 7.39–7.18 (*m*, 4H), 7.00 (d, *J* = 1.9 Hz, 1H), 4.24 (*q, J* = 7.1 Hz, 2H), 2.42 (*s*, 3H), 1.26 (*t, J* = 7.1 Hz, 3H).

#### Ethyl 1-phenyl-1H-pyrazole-5-carboxylate (Compound 12b)

Purification by flash column chromatography on silica gel using 0.2% triethylamine and 2% ethyl acetate in petroleum ether afforded the isolation of desired product as a yellow oil in 43% yield. ^1^H NMR (300 MHz, Chloroform-*d*) δ 7.69 (d, *J* = 2.0 Hz, 1H), 7.49–7.36 (*m*, 5H), 7.03 (d, *J* = 2.0 Hz, 1H), 4.24 (*q, J* = 7.1 Hz, 2H), 1.24 (*t, J* = 7.1 Hz, 3H).

#### Ethyl 1H-pyrazole-5-carboxylate (Compound 12c)

The product was obtained as a dark brown solid in 98% yield. ^1^H NMR (300 MHz, Chloroform-*d*) δ 7.75 (d, *J* = 9.9 Hz, 1H), 6.86 (d, *J* = 2.1 Hz, 1H), 4.42 (*qd, J* = 7.1, 1.0 Hz, 2H), 1.41 (*t*, *J* = 7.1 Hz, 3H).

#### Ethyl 1–(4-chlorophenyl)-1H-pyrazole-5-carboxylate(Compound 12d)

The product was purified by flash chromatography using 2% ethyl acetate in petroleum ether and it was obtained as a light yellow powder in 23% yield. ^1^H NMR (400 MHz, Chloroform-*d*) δ 7.69 (d, *J* = 2.0 Hz, 1H), 7.45–7.41 (*m*, 2H), 7.40–7.36 (*m*, 2H), 7.03 (d, *J* = 2.0 Hz, 1H), 4.26 (*q*, *J* = 7.1 Hz, 2H), 1.28 (*t*, *J* = 7.1 Hz, 3H).

#### Ethyl 1–(4-chlorophenyl)-1H-pyrazole-3-carboxylate(Compound 13d)

The product was obtained as a light yellow powder in 10% yield. ^1^H NMR (400 MHz, cdcl_3_) δ 7.90 (d, *J* = 2.5 Hz, 1H), 7.70 (d, *J* = 9.0 Hz, 2H), 7.55–7.40 (*m*, 2H), 6.99 (d, *J* = 2.5 Hz, 1H), 4.44 (*q*, *J* = 7.1 Hz, 2H), 1.42 (*t*, *J* = 7.1 Hz, 3H).

#### Ethyl 1–(4-bromophenyl)-1H-pyrazole-5-carboxylate(Compound 12e)

The product was purified by flash column chromatography on silica gel using 1.7% triethylamine and 3.4% of ethyl acetate in petroleum ether. The product was obtained as light yellow powder in 35% yield. ^1^H NMR (300 MHz, Chloroform-d) δ 7.69 (d, *J* = 2.0 Hz, 1H), 7.64–7.53 (*m*, 2H), 7.38–7.26 (*m*, 2H), 7.03 (d, *J* = 2.0 Hz, 1H), 4.26 (*q, J* = 7.2 Hz, 2H), 1.28 (*t, J* = 7.1 Hz, 3H).

#### Ethyl 1–(4-bromophenyl)-1H-pyrazole-3-carboxylate(Compound 13e)

The product was obtained as light yellow powder in 23% yield. ^1^H NMR (300 MHz, Chloroform-*d*) δ 7.91 (dd, *J* = 2.5, 0.6 Hz, 1H), 7.70–7.51 (*m*, 4H), 7.00 (dd, *J* = 2.6, 0.6 Hz, 1H), 4.59–4.34 (*m*, 2H), 1.42 (*td*, *J* = 7.1, 0.6 Hz, 3H).

#### Ethyl 1–(4-(tert-butyl)phenyl)-1H-pyrazole-5-carboxylate(Compound 12f)

The product was purified by flash column chromatography on silica gel using 0.2% triethylamine and 2% ethyl acetate in petroleum ether as eluent. The product was obtained as a yellow oil in 42% yield. ^1^H NMR (300 MHz, Chloroform-*d*) δ 7.67 (d, *J* = 2.0 Hz, 1H), 7.51–7.41 (*m*, 2H), 7.41–7.29 (*m*, 2H), 7.01 (d, *J* = 2.0 Hz, 1H), 4.24 (*q*, *J* = 7.2 Hz, 2H), 1.36 (*s*, 9H), 1.24 (*t*, *J* = 7.1 Hz, 3H).

#### Ethyl 1–(4-(tert-butyl)phenyl)-1H-pyrazole-3-carboxylate(Compound 13f)

The product was obtained as a yellow oil in 46% yield. ^1^H NMR (300 MHz, Chloroform-*d*) δ 7.90 (d, *J* = 2.5 Hz, 1H), 7.66 (d, *J* = 8.9 Hz, 2H), 7.47 (d, *J* = 8.8 Hz, 2H), 6.98 (d, *J* = 2.5 Hz, 1H), 4.44 (*q*, *J* = 7.1 Hz, 2H), 1.42 (*t*, *J* = 7.1 Hz, 3H), 1.35 (*s*, 9H).

#### Ethyl 1–(4-fluorophenyl)-1H-pyrazole-5-carboxylate(Compound 12g)

The product was purified by flash column chromatography on silica gel using 0.2% triethylamine and 2% ethyl acetate in petroleum ether. The product was obtained as a yellow powder in 47% yield. ^1^H NMR (300 MHz, Chloroform-*d*) δ 7.68 (d, *J* = 2.0 Hz, 1H), 7.50–7.34 (*m*, 2H), 7.21–7.07 (*m*, 2H), 7.02 (d, *J* = 2.0 Hz, 1H), 4.25 (*q*, *J* = 7.1 Hz, 2H), 1.27 (*t*, *J* = 7.1 Hz, 3H).

#### Ethyl 1–(4-fluorophenyl)-1H-pyrazole-3-carboxylate(Compound 13g)

The product was obtained as a yellow powder in 20% yield. ^1^H NMR (300 MHz, Chloroform-*d*) δ 7.87 (d, *J* = 2.5 Hz, 1H), 7.82–7.62 (*m*, 2H), 7.23–7.10 (*m*, 2H), 6.99 (d, *J* = 2.5 Hz, 1H), 4.44 (*q*, *J* = 7.1 Hz, 2H), 1.42 (*t*, *J* = 7.1 Hz, 3H).

#### Ethyl 1-cyclohexyl-1H-pyrazole-5-carboxylate (Compound 12h)

Purification by flash column chromatography on silica gel using 0.2% triethylamine and 2% ethyl acetate in petroleum ether allowed the isolation of the desired product in 39% yield as a colorless oil. ^1^H NMR (300 MHz, Methanol-*d*_4_) δ 7.52 (d, *J* = 2.0 Hz, 1H), 6.86 (d, *J* = 2.0 Hz, 1H), 5.16 (dq, *J* = 10.4, 5.4, 4.1 Hz, 1H), 4.36 (*q*, *J* = 7.1 Hz, 2H), 2.09–1.69 (*m*, 7H), 1.58–1.15 (*m*, 6H).

#### Ethyl 1-cyclohexyl-1H-pyrazole-3-carboxylate (Compound 13h)

The product was obtained as a yellow oil in 39% yield. ^1^H NMR (300 MHz, Methanol-*d*_4_) δ 7.75 (d, *J* = 2.5 Hz, 1H), 6.78 (d, *J* = 2.4 Hz, 1H), 4.37 (*q*, *J* = 7.2 Hz, 2H), 4.32–4.09 (*m*, 1H), 2.12 (d, *J* = 14.1 Hz, 2H), 1.99–1.63 (*m*, 8H), 1.39 (*t*, *J* = 7.2 Hz, 3H).

#### General procedure for the synthesis of1-R-pyrazole-5-carboxylic acid

To a solution of the proper ethyl 1R-pyrazole-5-carboxylate (0.203 mmol) solubilized in ethanol (0.6 ml) was added sodium hydroxide 6 M (36 μl, 1.2 mmol). Reaction mixture was stirred at 80 C for 2 h. The solvent was evaporated under reduced pressure, water was added and the residue acidified with HCl 1 M until pH 1–2. The precipitate was filtrated and washed with petroleum ether.

#### 1-(p-tolyl)-1H-pyrazole-5-carboxylic acid (Compound 4a)

The product was obtained as a white solid in 66% yield. ^1^H NMR (300 MHz, Methanol-*d*_4_) δ 7.70 (d, *J* = 2.0 Hz, 1H), 7.29 (*s*, 4H), 7.04 (d, *J* = 2.0 Hz, 1H), 2.43 (*s*, 3H).^13^ C NMR (101 MHz, Methanol-*d*_4_) δ 161.70, 140.44, 140.01, 139.29, 135.67, 130.15, 126.95, 113.42, 21.17. HRMS (ESI): calculated for C_11_H_9_O_2_N_2_ [M-H] 201.0670 found 201.06691.

#### 1-phenyl-1H-pyrazole-5-carboxylic acid (Compound 4b)

The product was obtained as a yellow powder in 78% yield. ^1^H NMR (300 MHz, Methanol-*d*_4_) δ 7.67 (d, *J* = 2.0 Hz, 1H), 7.50–7.33 (*m*, 5H), 7.02 (d, *J* = 2.0 Hz, 1H).^13^ C NMR (101 MHz, Methanol-*d*_4_) δ 161.62, 141.73, 140.64, 135.65, 129.74, 129.65, 127.16, 113.60. HRMS (ESI): calculated for C_10_H_7_O_2_N_2_ [M-H] 187.0513 found 187.05122.

#### 1H-pyrazole-5-carboxylic acid (Compound 4c)

The product was obtained as a pearl solid in 40% yield. ^1^H NMR (300 MHz, Methanol-*d*_4_) δ 7.69 (d, *J* = 2.6 Hz, 1H), 6.81 (d, *J* = 2.7 Hz, 1H).^13^ C NMR (101 MHz, Methanol-*d*_4_) δ 164.62, 142.92, 133.87, 109.03. HRMS (ESI): calculated for C_4_H_3_O_2_N_2_ [M-H] 111.0200 found 111.0200.

#### 1–(4-chlorophenyl)-1H-pyrazole-5-carboxylic acid (Compound 4d)

The product was obtained as a white powder in 77% yield. ^1^H NMR (300 MHz, Methanol-*d*_4_) δ 7.72 (d, *J* = 2.0 Hz, 1H), 7.54–7.37 (*m*, 4H), 7.05 (d, *J* = 2.0 Hz, 1H).^13^ C NMR (101 MHz, Methanol-*d*_4_) δ 161.74, 142.78, 140.98, 140.42, 135.40, 129.72, 128.69, 113.74. HRMS (ESI): calculated for C_10_H_6_ClO_2_N_2_ [M-H] 221.0123 found 221.01221.

#### 1–(4-chlorophenyl)-1H-pyrazole-3-carboxylic acid (Compound 5d)

The product was obtained as a white powder in 87% yield. ^1^H NMR (300 MHz, Methanol-*d*_4_) δ 8.32 (d, *J* = 2.5 Hz, 1H), 7.94–7.77 (*m*, 2H), 7.62–7.43 (*m*, 2H), 6.99 (d, *J* = 2.5 Hz, 1H).^13^ C NMR (101 MHz, Methanol-*d*_4_) δ 165.07, 146.75, 139.71, 134.20, 130.72, 130.63, 122.22, 111.38. HRMS (ESI): calculated for C_10_H_6_ClO_2_N_2_ [M-H] 221.0123 found 221.01237.

#### 1–(4-bromophenyl)-1H-pyrazole-5-carboxylic acid (Compound 4e)

The product was obtained as a light yellow powder in 91% yield. ^1^H NMR (300 MHz, Methanol-*d*_4_) δ 7.73 (d, *J* = 2.0 Hz, 1H), 7.69–7.58 (*m*, 2H), 7.42–7.30 (*m*, 2H), 7.07 (d, *J* = 2.0 Hz, 1H).^13^ C NMR (101 MHz, Methanol-*d*_4_) δ 161.48, 141.03, 140.85, 135.66, 132.77, 128.99, 123.32, 113.93. HRMS (ESI): calculated for C_10_H_6_BrO_2_N_2_ [M-H] 264.9618 found 264.96170.

#### 1–(4-bromophenyl)-1H-pyrazole-3-carboxylic acid (Compound 5e)

The product was obtained as a light yellow powder in 73% yield.^1^H NMR (300 MHz, Methanol-*d*_4_) δ 8.32 (d, *J* = 2.6 Hz, 1H), 7.84–7.75 (*m*, 2H), 7.71–7.63 (*m*, 2H), 6.99 (d, *J* = 2.6 Hz, 1H).^13^ C NMR (101 MHz, Methanol-*d*_4_) δ 165.07, 146.79, 140.17, 133.75, 130.59, 122.47, 121.91, 111.41. HRMS (ESI): calculated for C_10_H_6_BrO_2_N_2_ [M-H] 264.9618 found 264.96161.

#### 1–(4-(tert-butyl)phenyl)-1H-pyrazole-5-carboxylic acid(Compound 4f)

The product was obtained as a cream powder in 47% yield. ^1^H NMR (300 MHz, Methanol-*d*_4_) δ 7.70 (d, *J* = 2.1 Hz, 1H), 7.58–7.47 (*m*, 2H), 7.40–7.27 (*m*, 2H), 7.02 (d, *J* = 2.1 Hz, 1H), 1.38 (*s*, 9H).^13^ C NMR (101 MHz, Methanol-*d*_4_) δ 162.45, 153.17, 140.64, 139.45, 136.48, 126.78, 113.34, 111.64, 35.82, 31.95. HRMS (ESI): calculated for C_14_H_15_O_2_N_2_ [M-H] 243.1139 found 243.11357.

#### 1–(4-(tert-butyl)phenyl)-1H-pyrazole-3-carboxylic acid(Compound 5f)

The product was obtained as a cream powder in 64% yield. ^1^H NMR (300 MHz, Methanol-*d*_4_) δ 8.19 (d, *J* = 2.5 Hz, 1H), 7.82–7.64 (*m*, 2H), 7.62–7.44 (*m*, 2H), 6.91 (d, *J* = 2.4 Hz, 1H), 1.36 (d, *J* = 2.3 Hz, 9H).^13^ C NMR (101 MHz, Methanol-*d*_4_) δ 167.56, 151.63, 149.16, 138.90, 129.94, 127.44, 120.42, 110.52, 35.45, 31.70. HRMS (ESI): calculated for C_14_H_15_O_2_N_2_ [M-H] 243.1139 found 243.11377.

#### 1–(4-fluorophenyl)-1H-pyrazole-5-carboxylic acid (Compound 4g)

The product was obtained as a yellow powder in 93% yield. ^1^H NMR (300 MHz, Methanol-*d*_4_) δ 7.67 (d, *J* = 2.0 Hz, 1H), 7.47–7.34 (*m*, 2H), 7.24–7.10 (*m*, 2H), 7.02 (d, *J* = 2.0 Hz, 1H).^13^ C NMR (101 MHz, Methanol-*d*_4_) δ 165.16, 161.49, 140.73, 137.94, 135.77, 129.37, 129.28, 116.47, 116.24, 113.65. HRMS (ESI): calculated for C_10_H_6_FO_2_N_2_ [M-H] 205.0419 found 205.04185.

#### 1–(4-fluorophenyl)-1H-pyrazole-3-carboxylic acid (Compound 5g)

The product was obtained as a yellow powder in 36% yield. ^1^H NMR (400 MHz, Methanol-*d*_4_) δ 8.25 (d, *J* = 2.6 Hz, 1H), 7.90–7.76 (*m*, 2H), 7.31–7.18 (*m*, 2H), 6.96 (d, *J* = 2.5 Hz, 1H).^13^C NMR (101 MHz, Methanol-*d*_4_) δ 164.44, 161.99, 146.52, 137.49, 137.46, 130.78, 123.02, 122.93, 117.44, 117.21, 111.23. HRMS (ESI): calculated for C_10_H_6_FO_2_N_2_ [M-H] 205.0419 found 205.04185.

#### 1-cyclohexyl-1H-pyrazole-5-carboxylic acid (Compound 4h)

The product was obtained as a white powder in 86% yield. ^1^H NMR (400 MHz, Methanol-*d*_4_) δ 7.49 (dt, *J* = 2.0, 1.0 Hz, 1H), 6.83 (d, *J* = 2.0 Hz, 1H), 5.19 (ddd, *J* = 11.1, 7.6, 4.2 Hz, 1H), 1.92 (dddd, *J* = 21.5, 11.3, 5.6, 2.8 Hz, 6H), 1.78–1.70 (*m*, 1H), 1.53–1.39 (*m*, 2H), 1.35–1.18 (*m*, 1H).^13^C NMR (101 MHz, Methanol-*d*_4_) δ 162.40, 138.70, 133.53, 112.26, 60.46, 34.22, 26.78, 26.52. HRMS (ESI): calculated for C_10_H_13_O_2_N_2_ [M-H] 193.0983 found 193.09834.

#### 1-cyclohexyl-1H-pyrazole-3-carboxylic acid (Compound 5h)

The product was obtained as a white powder in 80% yield. ^1^H NMR (300 MHz, Methanol-*d*_4_) δ 7.72 (d, *J* = 2.4 Hz, 1H), 6.75 (d, *J* = 2.4 Hz, 1H), 4.21 (tt, *J* = 11.5, 3.9 Hz, 1H), 2.16–2.01 (*m*, 2H), 2.00–1.63 (*m*, 6H), 1.60–1.17 (*m*, 2H).^13^C NMR (101 MHz, Methanol-*d*_4_) δ 165.44, 144.13, 130.30, 109.26, 63.39, 34.37, 26.41, 26.30. HRMS (ESI): calculated for C_10_H_13_O_2_N_2_ [M-H] 193.0983 found 193.09834.

## Conclusions

The emergence of multidrug-resistant bacteria is considered one of the biggest health insults of the next decades. Targeting non-essential biochemical pathway such as the bacterial cysteine biosynthesis has recently attracted attention as a possible new antibacterial strategy. In particular, inhibition of *O*-acetylserine sulphhydrylase (OASS) has been proposed as a fruitful strategy to prevent the synthesis of cysteine. In addition to delivering the most potent inhibitor of OASS disclosed so far^34^, the aim of this work was to identify a novel chemical tool for the inhibition of OASS that would escape from the cyclopropane scaffold, as its synthetic restrictions somehow limited the synthesis of analogues. To this end, we have coupled computational aided drug design methodologies with a ligand-based medicinal chemistry approach. Although the small set of compounds tested against recombinant enzyme from *Salmonella*, we successfully identified two small fragments (1H-pyrrole-2-carboxylic acid and thiazole-2-carboxylic acid) with promising StOASS inhibitory characteristics, which will serve as chemical templates for improved cysteine biosynthesis blockers. In addition to these promising biological characteristics, it must be considered that these heterocyclic carboxylic acids have physicochemical properties that indicate potential for penetration through the Gram-negative bacterial cell wall, and are endowed with considerable drug-like characteristics, as they do not show any violation of the four physicochemical characteristics defined by the Lipinski rule of five. The chemical manipulation and biological evaluation in cell of these inhibitors are currently ongoing in our laboratories.
